# Meta-Analysis of a Complex Network of Non-Pharmacological Interventions: The Example of Femoral Neck Fracture

**DOI:** 10.1371/journal.pone.0146336

**Published:** 2016-01-06

**Authors:** Jonathan Mosseri, Ludovic Trinquart, Rémy Nizard, Philippe Ravaud

**Affiliations:** 1 INSERM U1153, Paris, France; 2 Assistance Publique-Hôpitaux de Paris, Hôpital Lariboisière, Service de Chirurgie orthopédique et traumatologique, Paris, France; 3 Université Paris Diderot, Paris, France; 4 Assistance Publique-Hôpitaux de Paris, Hôpital Hôtel-Dieu, Centre d’épidémiologie clinique, Paris, France; 5 French Cochrane Centre, Paris, France; 6 Université Paris Descartes–Sorbonne Paris cité, Paris, France; 7 Columbia University, Mailman School of Public Health, Department of Epidemiology, New York, United States of America; Harvard Medical School, UNITED STATES

## Abstract

**Background:**

Surgical interventions raise specific methodological issues in network meta-analysis (NMA). They are usually multi-component interventions resulting in complex networks of randomized controlled trials (RCTs), with multiple groups and sparse connections.

**Purpose:**

To illustrate the applicability of the NMA in a complex network of surgical interventions and to prioritize the available interventions according to a clinically relevant outcome.

**Methods:**

We considered RCTs of treatments for femoral neck fracture in adults. We searched CENTRAL, MEDLINE, EMBASE and ClinicalTrials.gov up to November 2015. Two reviewers independently selected trials, extracted data and used the Cochrane Collaboration’s tool for assessing the risk of bias. A group of orthopedic surgeons grouped similar but not identical interventions under the same node. We synthesized the network using a Bayesian network meta-analysis model. We derived posterior odds ratios (ORs) and 95% credible intervals (95% CrIs) for all possible pairwise comparisons. The primary outcome was all-cause revision surgery.

**Results:**

Data from 27 trials were combined, for 4,186 participants (72% women, mean age 80 years, 95% displaced fractures). The median follow-up was 2 years. With hemiarthroplasty (HA) and total hip arthroplasty (THA) as a comparison, risk of surgical revision was significantly higher with the treatments unthreaded cervical osteosynthesis (OR 8.0 [95% CrI 3.6–15.5] and 5.9 [2.4–12.0], respectively), screw (9.4 [6.0–16.5] and 6.7 [3.9–13.6]) and plate (12.5 [5.8–23.8] and 7.8 [3.8–19.4]).

**Conclusions:**

In older women with displaced femoral neck fractures, arthroplasty (HA and THA) is the most effective treatment in terms of risk of revision surgery.

**Systematic Review Registration:**

PROSPERO no. CRD42013004218.

**Level of Evidence:**

Network Meta-Analysis, Level 1.

## Introduction

Network meta-analysis (NMA) allows for comparing the relative benefits associated with multiple interventions used for the same disease [1]. Surgical interventions may raise specific methodological issues in NMA. In fact, they are usually multi-component interventions, which are frequently poorly described and assessed with a wide range of outcome measures in primary trials [2]. This diversity may result in complex networks of randomized controlled trials (RCTs), with multiple groups and sparse connections. We illustrate these potential issues with an NMA of treatments for femoral neck fractures.

Proximal femur fractures are among the most common injuries in trauma [3]. In 2000, an estimated 1.6 million hip fractures occurred worldwide [4] and this incidence is expected to increase to more than 6 million by the year 2050 [5–8]. Most hip fractures occur in older people with an average age of about 80 years [9]

Two major therapeutic orientations can be considered: osteosynthesis and arthroplasty. Regarding arthroplasty, several possibilities exist for the surgical approach, the type of implant, and the choice of cementing the femoral stem.

Which surgical treatment options for femoral neck fractures are best for which patients has been controversial for more than 50 years [10–13]. The best operative management option may depend on the type of patient (older or younger adults) and the type of fracture (nondisplaced or displaced fractures). Although most patients undergo arthroplasty, there are still unwarranted practice variations in the treatment methods of displaced femoral neck fractures in older adults, and several studies have shown a lack of consensus [14–18]. Multiple treatments have been compared in head-to-head RCTs. However, not all treatment options have been compared against each other. In addition, conventional systematic reviews and meta-analyses (MAs) have been performed [3, 19–29]. However, uncertainty remains regarding the best therapeutic option, especially concerning the risk of revision surgery [24]. We lack consensus on how best to treat femoral neck fractures with the primary intention of avoiding reoperation and additional risks [30]. As well, the estimated risk of mortality for patients with hip fracture at 1 year was about 20% (95% CI 16–24%) as compared with controls (11% [95% CI 8–15%]) [28, 31].

The objectives of this study were to illustrate the applicability of the NMA in a complex network of surgical interventions and to prioritize the available interventions according to a clinically relevant outcome. We performed an NMA of RCTs in adult patients with an intracapsular femoral neck fracture, evaluating all surgical therapeutic procedures. Our primary outcome was rate of revision surgery, regardless of cause.

## Methods

This systematic review was registered on PROSPERO (no. CRD42013004218). This report follows the recommendations of the PRISMA extension statement for systematic reviews incorporating network meta-analyses (Table A in S1 File) [32, 33].

### Criteria for considering trials

We searched for reports of RCTs in any language comparing at least one surgical treatment for intracapsular femoral neck fracture to another surgical technique in all adults (≥ 18 years old) without any restriction on age, preoperative autonomy, gender or comorbidities. All degrees of fracture displacement were eligible. We considered all available treatments, without any restriction. We selected only trials that reported our primary outcome.

### Search strategy

First, we searched the Cochrane Database of Systematic Reviews, the Database of Abstracts of Reviews of Effects and MEDLINE for relevant systematic reviews and MAs of RCTs on the topic from inception to November 2015. From each selected review, we first collected the search equations used, to optimize our own search strategy. Second, we listed the included trials.

Next, we searched CENTRAL, MEDLINE, and EMBASE for reports of RCTs from inception to November 2015, with no restriction on date or language. The search equation combined free-text words and keywords (Text A in S2 File). We used the methodological filters designed by the Cochrane collaboration to facilitate the identification of randomized trials (“Highly Sensitive Search Strategy” filter for MEDLINE and the filter designed by the UK Cochrane Center for EMBASE).

We manually searched for trial results published by the American Academy of Orthopedic Surgeons (AAOS) from 2011 to 2013 and searched ClinicalTrials.gov for reports up to November 2015.

### Selection of trials

Two investigators independently screened titles and abstracts, then selected full-text articles. Discrepancies were discussed to obtain a consensus. The reasons for excluding trials or publications were documented (Table B in S2 File).

### Data extraction and management

Two reviewers independently used a standardized extraction form to extract data on patient and treatment characteristics and to assess the methodological quality of trials. If required, trial authors were contacted to obtain missing information.

### Assessment of risk of bias in selected trials

Two specially trained reviewers independently assessed the risk of bias in the selected RCT reports using the Cochrane Collaboration Risk of Bias tool [34]. We assessed sequence generation, allocation concealment, blinding and incomplete outcome data. Each domain was evaluated for low, high or unclear risk of bias. We assessed the risk of bias due to lack of blinding for each outcome separately. The surgical revision outcome is not objective enough to consider that knowledge of the treatment does not alter its assessment. Mortality is an objective outcome: the lack of blinding does not change its evaluation.

### Classification of interventions

The description of the interventions by the original trial authors was extracted from each eligible report. A small group of orthopedic surgeons trained in epidemiology assessed the similarities of these interventions and grouped similar but not identical interventions under the same therapeutic class (Table C in S2 File). We defined homogeneous therapeutic classes by a consensus process. Regarding osteosynthesis, we distinguished 3 classes of treatment: screw, unthreaded cervical osteosynthesis (UCO; defined as unthreaded implants inserted into the femoral neck without an extramedullary component) and plate (all extramedullary implants). Regarding arthroplasty, we distinguished HA and total hip arthroplasty (THA). If required, the trial authors were contacted to obtain clarification about the specific implant devices or techniques.

### Geometry of networks of trials

We produced graphs for 2 networks of trials. Each node represented an intervention and each edge a randomized comparison of 2 interventions. The first network included all trials and nodes corresponding to interventions as labeled by the original trial authors. The second network included trials contributing to the primary outcome and after grouping interventions as described above. We excluded two-arm trials comparing interventions within the same class (eg, a trial comparing unipolar and bipolar HA). A three-arm trial involving 2 interventions within the same class was analyzed as a two-arm trial by adding the outcome data for the arms that were grouped together. We excluded trials lacking a full description of interventions (eg, arthroplasty or osteosynthesis) and trials assessing a mixture of interventions (eg, screw or pin). We also excluded trials that were not connected to the network.

We analyzed the network geometry by graphically examining the connections between interventions [35]. We examined which of the considered treatments (nodes) were compared head to head in RCTs, which of the considered treatments were connected indirectly by one or more “common comparators,” and the level of evidence informing each comparison.

### Data synthesis

We synthesized data from the second network (ie, RCTs contributing to the primary outcome after grouping interventions). Data were analyzed on an intent-to-treat basis: the analysis was based on the total number of randomly assigned participants. For missing outcome data, we used a conservative approach and imputed outcomes for the missing participants assuming that they did not experience the outcome (revision surgery). The treatment effect measure was the odds ratio (OR).

First, we performed pairwise random-effects MAs by synthesizing trials that compared the same interventions. Statistical heterogeneity was assessed by visual inspection of forest plots and by calculating the I^2^ statistic and between-trial variance τ^2^.

Second, we performed a random-effects NMA within a Bayesian framework [36]. Screw treatment was chosen as the reference treatment across the network because it had been compared with the highest number of other treatments. We assumed homogeneity of the between-trial variance across all comparisons and consistency between direct and indirect evidence. We chose non-informative prior distributions for contrasts versus screw treatment (basic parameters *θ*~N(0,10^5^)) and a vague prior for the between-trial standard deviation (*τ*~U(0,10)). In a sensitivity analysis, we used an alternative prior distribution, a vague Gamma prior on the precision (1/*τ*^2^~Gamma(0.001,0.001)) and we found similar results. We reported the posterior mean ORs (and associated 95% credible intervals [95% CrIs]) for all comparisons between treatments in the network. We derived absolute treatment effects (absolute risk of event associated with each treatment). We also reported measures of ranking between treatments (posterior mean ranks and associated 95% CrIs).

To assess inconsistency, we fitted an inconsistency model whereby each of the contrasts for which direct evidence was available was estimated without assuming consistency [37]. Comparison of the model fit (using the posterior deviance and the Deviance Information Criterion [DIC]) between the consistency and inconsistency models provided an “omnibus” test of consistency. We also assessed inconsistency locally by using the node-splitting method [38]. Finally, we assessed non-transitivity by comparing the distribution of potential effect modifiers across treatment comparisons (publication year, follow-up duration, mean age, proportion of females and patients with nondisplaced fracture).

To further validate the grouping of interventions (subnodes) into classes (parent nodes), we also performed 2 NMAs on the connected network before grouping (ie, subnodes corresponding to interventions as labeled by the original trial authors). First, we assumed that subnodes were unrelated (independent). Second, we assumed that all subnodes belonging to a common parent node were identical (fixed nodes). We compared the model fit of the independent-subnodes model to that of the fixed-nodes model, consistency being assumed in both cases [39].

Analyses involved Meta-analyst for pairwise MAs and R 3.0.2 (R Development Core Team, Vienna, Austria) and WinBUGS v1.4.3 (Imperial College and MRC, London, UK) for NMAs.

## Results

### Selection of trials

**Fig 1:**
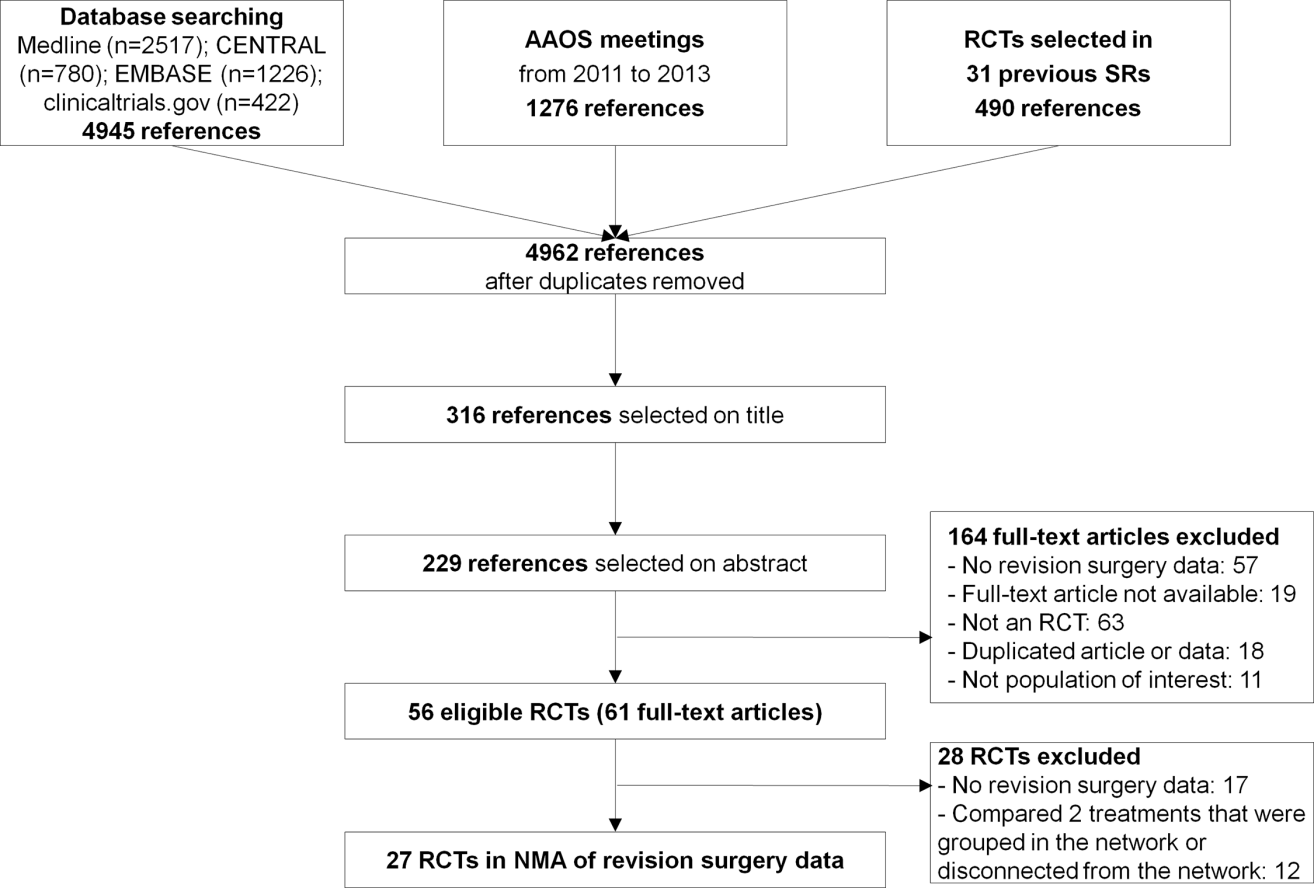
Flow diagram of the study selection. RCT, randomized controlled trial; SR, systematic review; MA, meta-analysis.

Fig 1 shows the flowchart for the selection of RCT reports. A total of 56 trials (61 articles) with 9,977 patients were eligible. We contacted the authors of 47 trials to clarify the eligibility of trial reports and received 15 responses; 3 provided primary outcome data. For 17 of the 56 RCTs, the reports did not provide data on revision surgery. Moreover, 22 trials could not contribute to the NMA because they compared 2 treatments that were grouped together in the network (eg, bipolar versus unipolar hemiarthoplasty [HA]) or because they were disconnected from the network (eg, cemented vs uncemented HA). Finally, we included 27 RCTs with 4,186 patients in the NMA of treatments for femoral neck fractures [40–66].

### Geometry of networks of trials

**Fig 2:**
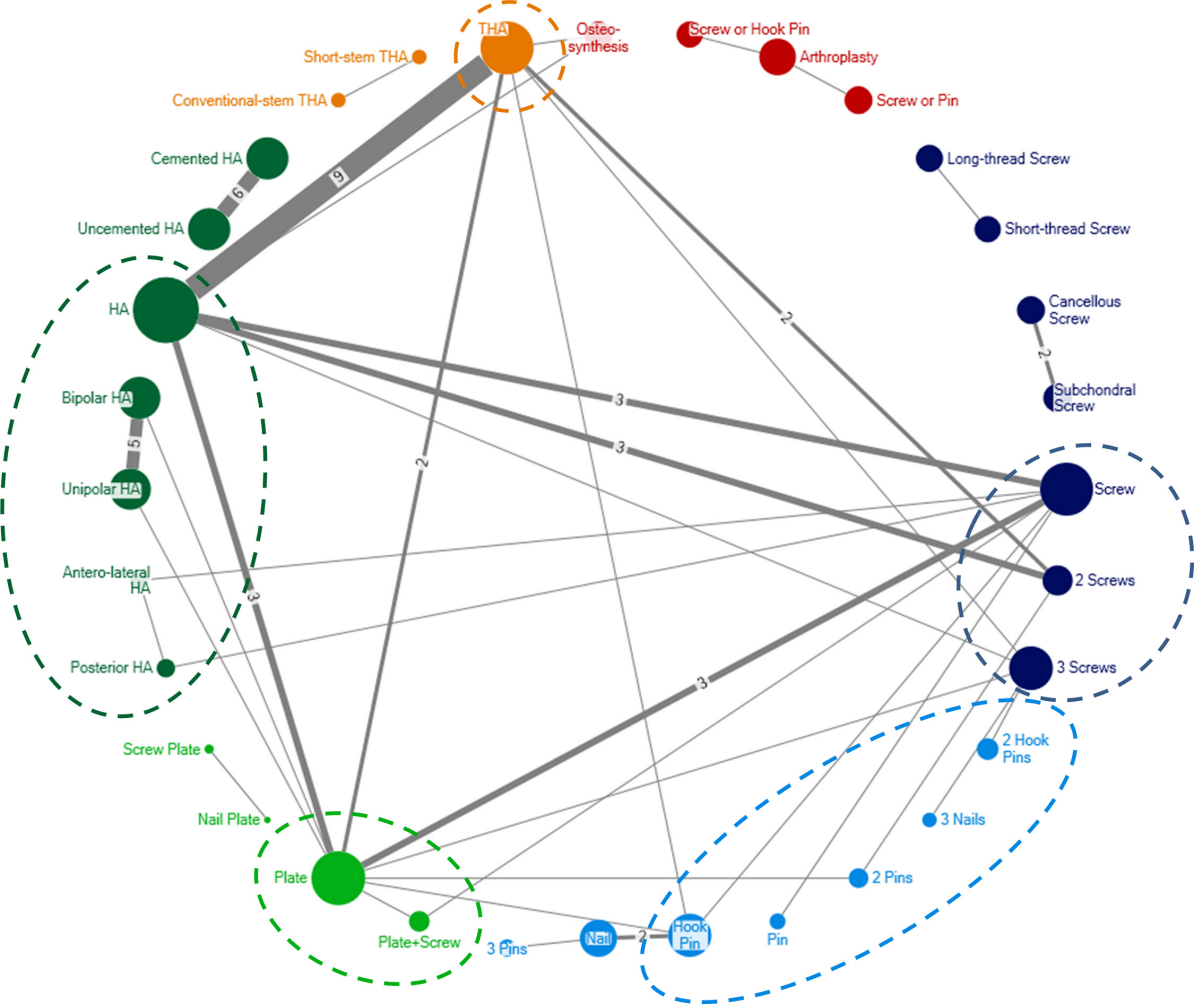
Network graph for 56 trials with interventions as labeled by the original trial authors. Each node represents a treatment and each edge a randomized comparison of 2 treatments. Each edge is labeled with the number of randomized comparisons, except when there was a single trial assessing the corresponding treatment comparison. Ellipses show treatments that were grouped. Dark blue: screw treatment; light blue: unthreaded cervical osteosynthesis; light green: plate treatment; dark green: hemiarthroplasty; orange; total hip arthroplasty; red: nonspecific interventions (arthroplasty or osteosynthesis) and mixture of interventions (screw or pin). HA hemiarthroplasty; THA total hip arthroplasty.

Fig 2 shows the complex network of the 56 eligible trials with interventions as labeled by the original trial authors (32 nodes, 37 edges). A total of 7 comparisons (13 trials) were not connected to the network. For 3 trials, one arm involved an intervention that was not specific enough.

Figure D in S2 File illustrates the evolution of the network of trials across time. Among the 17 trials published up to 1995, 13 (76%) compared osteosynthesis interventions against each other. The 34 trials corresponding to the year 2005 showed an increase in comparisons between osteosynthesis and arthroplasty (38%). The 56 trials up to 2015 showed an increase in comparisons between variations of arthroplasty.

**Fig 3:**
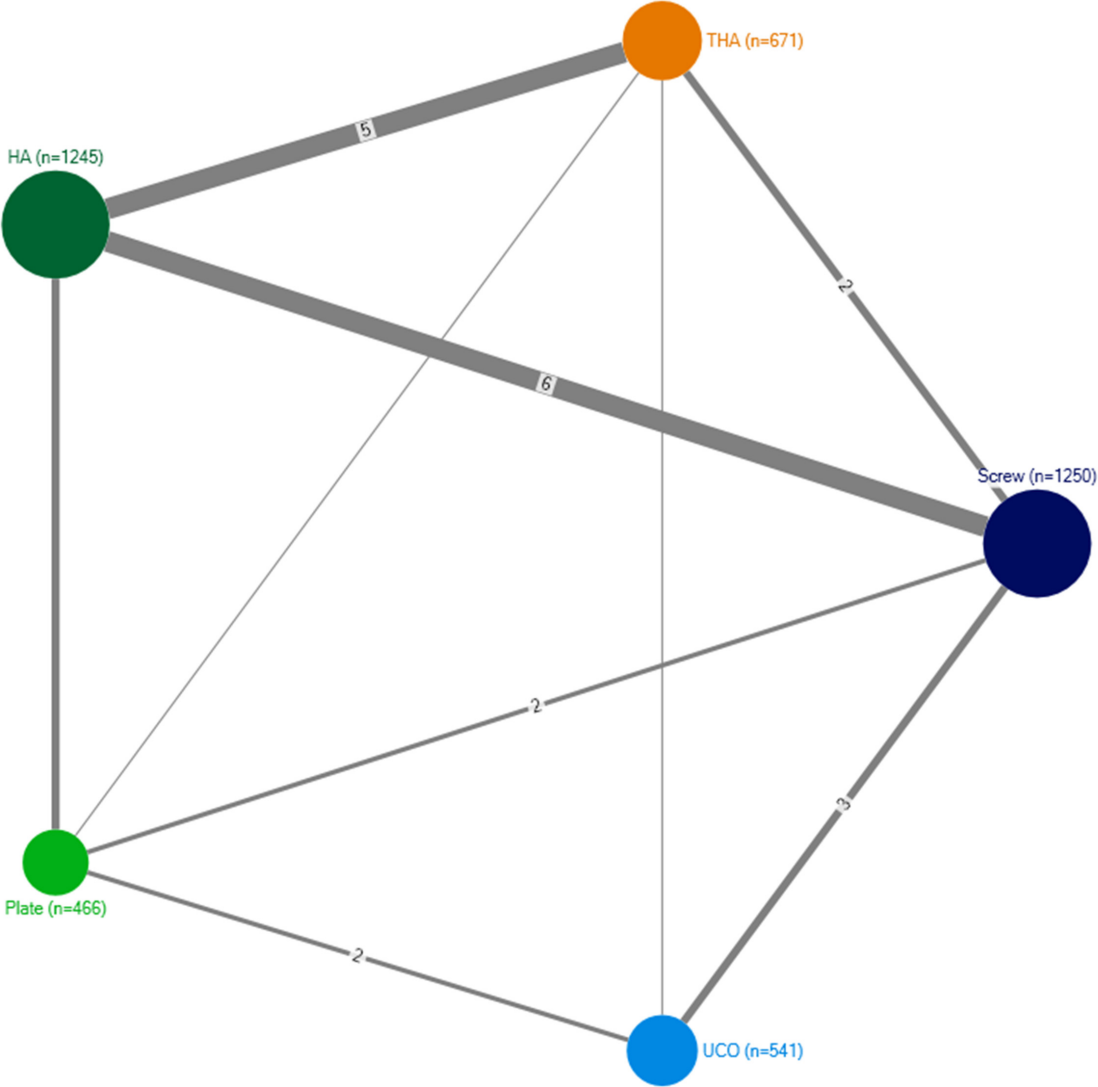
Network graph for 27 trials that reported the rate of revision surgery and after grouping interventions. Each node represents a treatment and each edge a randomized comparison of 2 treatments. Each edge is labeled with the number of randomized comparisons, except when there was a single trial assessing the corresponding treatment comparison. HA hemiarthroplasty; THA total hip arthroplasty; UCO unthreaded cervical osteosynthesis.

Fig 3 shows the network of 27 trials that reported the rate of revision surgery and after grouping interventions. The most common comparisons was screw treatment versus HA (n = 7) and THA versus HA (n = 7). Overall, 10 pairwise comparisons were possible between the 5 treatments, but one comparison (UCO versus HA) had no available trials.

### Characteristics of selected trials

Table 1 and Table E in S2 File show that the 27 trials included a median of 127 randomized patients (total 4,186) and were published between 1981 and 2015 (median: 2006). The median follow-up was 2 years (range 1 year to 5 years). The mean age of included patients was 80 years, and about 72% were women. We found a strong tendency to distinguish only displaced and non-displaced fractures, as recommended by some authors [67, 68]. Most trials (85%, n = 23) included only patients with displaced fractures (graded as Garden 3–4).

**Table 1:** Trial and patient characteristics for 27 randomized controlled trials of treatments for femoral neck fracture in adults.

	All RCTs (n = 27)	Screw vs. HA (n = 7)	HA vs. THA (n = 7)	Plate vs. HA (n = 3)	UCO vs. Screw (n = 3)	Screw vs. THA (n = 3)	Plate vs. Screw (n = 2)	UCO vs. Plate (n = 2)	UCO vs. THA (n = 1)	Plate vs. THA (n = 1)
Publication year, median [range]	2006 [1981–2015]	2003 [1981–2013]	2008 [2005–2013]	2007 [2001–2015]	2003 [1992–2006]	2003 [2000–2014]	2003 [1997–2012]	1992 [1988–1995]	1996	2007
Follow-up duration, months, median [min-max]	24 [12–60]	24 [24–60]	24 [12–60]	12 [12–36]	24 [24–24]	24 [24–60]	30 [24–36]	29 [24–33]	24	12
No of patients, median [min-max]	127 [32–455]	100 [32–455]	96 [41–252]	86 [56–280]	199 [180–278]	110 [100–285]	143 [60–225]	175 [127–222]	47	86
Proportion of women, mean [min-max]*	72% [0–96]	83% [71–96]	72% [51–81]	49% [0–69]	65% [37–82]	67% [54–75]	80% [79–82]	68%	77%	72%
Age, years, mean [min-max]*	80 [74–86]	82 [80–85]	79 [73–86]	77 [75–81]	80 [77–82]	80 [75–84]	80 [78–81]	78	79	74
Trials including both displaced and undisplaced fractures	4 (15%)	0	0	0	3 (100%)	0	0	1	0	0
Proportion of patients with undisplaced fracture, mean [min-max]	5% [0–100]	0% [0–0]	0% [0–0]	0% [0–0]	30% [28–35]	0% [0–0]	50% [0–100]	19% [0–59]	0%	0%

One three-arm trial compared HA vs. THA vs. Plate. HA hemiarthroplasty; THA total hip arthroplasty; UCO unthreaded cervical osteosynthesis

* unclear in 1 trial comparing UCO vs. Plate

### Assessment of risk of bias in selected trials

Figure F in S2 File shows the results of the assessment of risk of bias in selected RCT reports. In all, the risk of bias was high or unclear; only 11 reports (41%) described an adequate random sequence generation and 11 (41%) an adequately concealed treatment allocation.

### Comparison of treatments in terms of surgical revision

We directly compared available pairs of treatments in the selected RCTs using pairwise MA (Table 2, Table G in S2 File). The risk of surgical revision was significantly increased with screw or plate treatment as compared with HA and THA.

**Table 2:** Meta-analysis (MA) and network MA (NMA) for the 6 treatment groups in terms of risk of revision surgery.

THA	0.7 [0.4–1.4]	**6.7** **[3.9–13.6]**	**7.8 [3.8–19.4]**	**5.9** **[2.4–12.0]**
0.8 [0.3–2.2]	HA	**9.4** **[6.0–16.5]**	**12.5** **[5.8–23.8]**	**8.0** **[3.6–15.5]**
**10.2** **[3.1–33.1]**	**7.9** **[4.4–14.4]**	Screw	1.3 [0.6–2.3]	0.8 [0.4–1.4]
12.4 [0.7–232.1]	**7.8** **[3.5–17.4]**	1.0 [0.2–4.3]	Plate	0.7 [0.3–1.3]
2.8 [0.6–12.3]		0.9 [0.6–1.2]	0.6 [0.3–1.2]	UCO

HA hemiarthroplasty; THA total hip arthroplasty; UCO unthreaded cervical osteosynthesis

Treatments are reported in the diagonal. Data are odds ratios (ORs) with 95% confidence/credible intervals. Below the diagonal, results of the pairwise MAs are reported; ORs compare the row-defining treatment versus the column-defining treatment. Above the diagonal, results of the NMA are reported; ORs compare the column-defining treatment versus the row-defining treatment. Significant results are in bold and underscored.

Table 2 shows the results of the NMA. The risk of surgical revision was significantly greater with screw, plate or UCO treatment as compared with HA or THA. Between-trial heterogeneity was moderate (τ^2^ = 0.23). Mean (95% CrI) treatment rankings were as follows: HA, 1.1 [1–2]; THA, 1.9 [1–2]; UCO, 3.3 [3–5]; screw, 4.1 [3–5]; and plate, 4.7 [3–5]. Table 3 shows the absolute risk of surgical revision for each treatment. The risk of surgical revision was low with HA and THA and high with osteosynthesis.

**Table 3:** Absolute risk of surgical revision for each treatment.

Treatment	Absolute risk	95% credible interval
Total hip arthroplasty	6.4%	0.1–21.9%
Hemiarthroplasty	5.0%	0.1–18.2%
Unthreaded cervical osteosynthesis	25.0%	4.0–64.3%
Screw	29.1%	5.3–68.7%
Plate	32.3%	6.0–74.0%

Median follow-up time 2 years.

We found no evidence of global inconsistency in the network (Table H in S2 File). The consistency and inconsistency models had similar fit to the data (posterior mean residual deviance = 68.3 and 64.3 and DIC = 298.9 and 299.8, respectively). The node-splitting method showed no statistically significant consistency for any comparison with direct evidence available. When assessing clinical and methodological transitivity, all characteristics were similar across trials and comparisons (Table 1).

In a sensitivity analysis, the 2 NMA models of the connected network before grouping, assuming that subnodes were unrelated and assuming that all subnodes belonging to a common parent node were identical, provided similar results and had similar fit to the data (data not shown).

## Discussion

In this study, we illustrated the issues raised in NMA of surgical interventions and suggested potential solutions. We assessed the relative effectiveness of surgical treatments for intracapsular femoral neck fracture in 27 RCTs including 4,186 patients followed for a median of 2 years. Patients were mostly older women with displaced fractures. This NMA showed that among THA, HA, screw, plate and UCO treatments, arthroplasty (THA and HA) was associated with the lowest rate of surgical revision.

To our knowledge, this is the first NMA investigating all treatment options for femoral neck fracture. Several systematic reviews and MAs previously compared 2 interventions, but the conclusions were necessarily incomplete in terms of the best option because not all treatments had been compared against each other [19–26, 69]. By direct comparisons, we could assess only 9 comparisons of the 5 therapeutic classes considered. The NMA allowed for producing coherent estimates for all 15 possible pairwise comparisons, using all available evidence.

Current recommendations are for THA for patients with a displaced fracture who are able to walk independently, are not cognitively impaired and are medically fit for surgery [70]. In older adults and the oldest of the old population, the surgeon may choose a less invasive treatment, and prosthesis is not the unanimous choice in this population. Our findings suggest that arthroplasty may be a less disruptive option, even for patients older than 80, because of its lower risk of revision surgery as compared with osteosynthesis.

We did not find any difference between THA and HA in terms of reoperation. This finding differs from a systematic review concluding lower reoperation rates with THA than HA in older patients with displaced femoral neck fracture [29]. However, this synthesis included RCTs, quasi-randomized trials, and cohort studies; when restricted to RCTs, the MA yielded a combined risk ratio for THA versus HA of 1.1 (95% CI 0.4–3.0), which is consistent with our findings.

While the use of NMA has expanded exponentially, the method has been rarely applied to analysis of surgical interventions. In a review of 121 NMAs, 9% assessed surgical interventions and 8% both drug and non-drug interventions [71]. An area of specific concern with surgical interventions may be the definition of the network of evidence and its nodes. In fact, the complex nature of non-drug treatments may lead to complex networks of evidence, with multiple nodes but few connections. In our case study, the original network had 32 nodes but only 37 edges. Moreover, many reports of primary trials do not provide sufficient detail about the interventions.

Here, we classified similar but not identical interventions into therapeutic classes by using a consensus process involving a group of surgeons trained in clinical epidemiology. Interventions within these therapeutic classes were considered similar in terms of the risk of revision surgery. Moreover, sensitivity analyses, with models proposed for use in analyzing networks and subnodes defined by drug dose, supported the classification of interventions [39]. An alternative modeling could be a class effect network meta-analysis model, where one would assume that each intervention effect in the same therapeutic class come from a family of effects with a mean effect specific to the therapeutic class and between-intervention variability within class (assumed equal across all therapeutic classes)[72]. An additional comment regarding the definition of nodes in the network is that, in our systematic review, we included trials which assessed non-threaded implants. We acknowledge that the use of this operative option has become less frequent and the assessment of this option may appear as irrelevant based on the advances of implants and surgical techniques. However, these trials compared non-threaded implants to screws, plate, and THA, respectively. As a consequence, they provided additional information, through indirect evidence, for the estimation of the relative efficacy in these focal treatment comparisons of interest [73].

A second factor that could make the synthesis more complex is that non-drug treatments are usually assessed with a range of outcomes. Here, we chose all-cause surgical revision as a primary outcome because it appeared to be the most clinically relevant outcome in a predominantly senior population with no performance requirement and for whom any surgical intervention is a risk [30]. Advanced statistical methods have been described recently to model multiple correlated outcomes [74, 75]. Another methodological issue that may affect networks of surgical trials is the risk of bias in trials. Here, the methodological quality of the included trials was frequently unclear or high.

Finally, we have used a Bayesian approach in our network meta-analysis. Following current recommendations and practice, we have placed a flat normal prior on treatment effects and a Uniform prior on the between-trial standard deviation. We acknowledge that it may be questionable what prior distribution should be used for this standard deviation. In a sparse network, the small number of trials may not allow identifying the posterior distribution of the between-trial standard deviation precisely; this could result in implausibly high or low values for the between-trial heterogeneity. Turner et al. have recently used Cochrane meta-analyses to derive predictive distributions for the between-trial heterogeneity in a variety of settings according to the outcomes assessed and comparisons made [76, 77]. These predictive distributions could be used to approximate informative priors and give better estimates of the between-trial standard deviation.

In a population of predominantly older women with displaced femoral neck fracture, arthroplasty (THA and HA) was the most effective treatment for femoral neck fractures in terms of risk of revision surgery. HA may have an advantage as the fastest and least expensive surgery. Although our NMA investigated only one aspect of a significant issue that confronts surgeons, our finding may have appeal to surgeons who do not treat this population with arthroplasty. As well, this study illustrated the strengths of NMA of complex surgical interventions, for which both clinical and methodological considerations are required.

## Supporting Information

S1 File PRISMA checklist of items for a Systematic Review Involving a Network Meta-analysis (Table A). (DOCX)

S2 File Details about the Methods and Results. The Supporting Information S2 File includes search equations (Text A); reasons for exclusion of 28 trials (Table B); therapeutic classes defined by a consensus process (Table C); evolution of the network of the 56 trials with interventions as labeled by the original trial authors (Figure D); summary data for each trial and for each intervention group (Table E); risk of bias in 27 selected trials (Figure F); pairwise meta-analyses for revision surgery (Table G); and assessment of consistency between direct and indirect evidence (Table H).
(DOC)
