# Supplementary material for: Meta-Analysis of a Complex Network of Non-Pharmacological Interventions: The Example of Femoral Neck Fracture
Source: PLoS One. 2016 Jan 6;11(1):e0146336. doi: 10.1371/journal.pone.0146336 (PMC4703382; doi:10.1371/journal.pone.0146336)
Supplement: S2 File — The Supporting Information S2 File includes search equations (Text A); reasons for exclusion of 28 trials (Table B); therapeutic classes defined by a consensus process (Table C); evolution of the network of the 56 trials with interventions as labeled by the original trial authors (Figure D); summary data for each trial and for each intervention group (Table E); risk of bias in 27 selected trials (Figure F); pairwise meta-analyses for revision surgery (Table G); and assessment of consistency between direct and indirect evidence (Table H). (DOC) [file pone.0146336.s002.doc]

# Supporting Information S2 File

# Text A. Search equations

## PUBMED

| **Population** |
| --- |
| 1. ("hip"[MeSH Terms] OR "hip"[All Fields] OR "neck"[MeSH Terms] OR "neck"[All Fields] OR "collum"[All Fields] OR subcapital[All Fields] OR basicervical[All Fields] OR transcervical[All Fields] OR intracapsular[All Fields] OR "femur"[MeSH Terms] OR "femur"[All Fields] OR "femoral"[All Fields]) AND ("fractures, bone"[MeSH Terms] OR "fracture"[All Fields] OR "fractures"[All Fields] OR "fractured"[All Fields]) NOT (extracapsular[Title] OR trochanteric[Title] OR shaft[Title] OR intertrochanteric[Title] OR subtrochanteric[Title] OR pertrochanteric[Title] OR periprosthetic[Title] OR osteoarthrititis[Title]) |
| **Interventions** |
| 2. ("arthroplasty"[MeSH Terms] OR "arthroplasty"[All Fields] OR "hemiarthroplasty"[MeSH Terms] OR "hemiarthroplasty"[All Fields] OR "prosthetic replacement"[All Fields] OR (prosthetic[All Fields] AND "replacement"[All Fields]) OR "prosthesis implantation"[MeSH Terms] OR "prosthesis"[All Fields] OR "prostheses"[All Fields] OR "prostheses and implants"[MeSH Terms] OR "implants"[All Fields] OR "fracture fixation, internal"[MeSH Terms] OR "internal fracture fixation"[All Fields] OR ("internal"[All Fields] AND "fixation"[All Fields]) OR "internal fixation"[All Fields] OR "bone nails"[MeSH Terms] OR "nail"[All Fields] OR "nails"[All Fields] OR "pin"[All Fields] OR "pins"[All Fields] OR "screw"[All Fields] OR "screws" [All Fields] OR "bone screws"[MeSH Terms] OR "bone plates"[MeSH Terms] OR "plate"[All Fields] OR "plates"[All Fields] OR "nails"[MeSH Terms] OR "nails"[All Fields] OR "nail"[All Fields] OR "internal fixators"[MeSH Terms] OR ("internal"[All Fields] AND "fixators"[All Fields]) OR "internal fixators"[All Fields] OR ("internal"[All Fields] AND "fixator"[All Fields]) OR "internal fixator"[All Fields] OR conservative[All Fields] OR nonsurgical[All Fields] OR non-surgical[All Fields] OR non-operative[All Fields] OR "bed rest"[MeSH Terms] OR ("bed"[All Fields] AND "rest"[All Fields]) OR "bed rest"[All Fields] OR "traction"[MeSH Terms] OR "traction"[All Fields] OR plaster[All Fields]) |
| **Cochrane Highly Sensitive Search Strategy** |
| 3. (randomized controlled trial[pt] OR controlled clinical trial[pt] OR randomized[tiab] OR placebo[tiab] OR drug therapy[sh] OR randomly[tiab] OR trial[tiab] OR groups[tiab]) NOT (animals[mh] NOT humans[mh]) |
| **Equation: #1 AND #2 AND #3** |

## EMBASE

| **Population** |
| --- |
| 1. (hip OR neck OR collum OR subcapital OR basicervical OR transcervical OR intracapsular OR femur OR femoral OR 'hip'/exp OR 'neck'/exp OR 'femur'/exp) |
| 2. (fracture OR fractures OR fractured OR 'fracture'/exp) |
| 3. (extracapsular:ti OR trochanteric:ti OR shaft:ti OR intertrochanteric:ti OR subtrochanteric:ti OR pertrochanteric:ti OR periprosthetic:ti OR osteoarthrititis:ti) |
| **Interventions** |
| 4. (arthroplasty OR hemiarthroplasty OR “prosthetic replacement” OR (prosthetic AND replacement) OR prosthesis OR prostheses OR implants OR “internal fracture fixation” OR (internal AND fixation) OR “internal fixation” OR nail OR nails OR pin OR pins OR screw OR screws OR plate OR plates OR nails OR nail OR (internal AND fixators) OR “internal fixators” OR (internal AND fixator) OR “internal fixator” OR conservative OR nonsurgical OR non-surgical OR non-operative OR (bed AND rest) OR “bed rest” OR traction OR plaster OR 'arthroplasty'/exp OR 'implantation'/exp OR 'orthopedic prostheses, orthoses and implants'/exp OR 'prostheses and orthoses'/exp OR 'osteosynthesis'/exp OR 'bone nail'/exp OR 'bone screw'/exp OR 'bone plate'/exp OR 'bone nail'/exp OR 'internal fixator'/exp OR 'bed rest'/exp OR 'traction therapy'/exp) |
| **Methodological filter** |
| 5. (random$ OR factorial$ OR crossover$ OR cross over$ OR cross-over$ OR placebo$ OR doubl$ adj blind$ OR singl$ adj blind$ OR assign$ OR allocat$ OR volunteer$ OR 'crossover procedure'/exp OR 'double blind procedure'/exp OR 'randomized controlled trial'/exp OR 'single blind procedure'/exp) |
| **Equation: (#1 NOT #3) AND #2 AND #4 AND #5** |

## CENTRAL

| **Population** |
| --- |
| 1. ((hip OR neck OR collum OR subcapital OR basicervical OR transcervical OR intracapsular OR femur OR femoral OR hip OR neck OR femur) NOT (extracapsular:ti OR trochanteric:ti OR shaft:ti OR intertrochanteric:ti OR subtrochanteric:ti OR pertrochanteric:ti OR periprosthetic:ti OR osteoarthrititis:ti)) AND (fracture OR fractures OR fractured OR fractures,bone ) |
| **Interventions** |
| 2. (arthroplasty OR hemiarthroplasty OR “prosthetic replacement” OR (prosthetic AND replacement) OR prosthesis OR prostheses OR implants OR “internal fracture fixation” OR (internal AND fixation) OR “internal fixation” OR nail OR nails OR pin OR pins OR screw OR screws OR plate OR plates OR nails OR nail OR (internal AND fixators) OR “internal fixators” OR (internal AND fixator) OR “internal fixator” OR conservative OR nonsurgical OR non-surgical OR non-operative OR (bed AND rest) OR “bed rest” OR traction OR plaster OR arthroplasty OR hemiarthroplasty OR “prosthesis implantation” OR prostheses and implants OR “fracture fixation, internal” OR “bone nails” OR “bone screws” OR “bone plates” OR nails OR “internal fixators”  OR “bed rest” OR traction) |
| **Equation: #1 AND #2** |

**Table B. Reasons for exclusion of 28 trials**

| **Trial** | **Comparisons** | **Revision surgery outcome** | **Other reason for exclusion*** |
| --- | --- | --- | --- |
| Svenningsen 1984 | Plate vs. screw | NR |  |
| Madsen 1987 | Plate vs. screw | NR |  |
| Alberts 1989 | 3 nails vs. 3 screws | NR |  |
| Skinner 1989 | HA vs. plate vs. THA | NR |  |
| Olerud 1991 | 2 pins vs. 2 screws | NR |  |
| Sorensen 1992 | Plate vs. screw | NR |  |
| Bonke 1999 | HA vs. THA | NR |  |
| van Dortmont 2000 | HA vs. screw | NR |  |
| Sonne-Holm 1982 | Cemented HA vs. uncemented HA | NR | Disconnected |
| Rehnberg 1989 | Cancellous screw vs. subchondral Screw | NR | Disconnected |
| Emery 1991 | Cemented HA vs. uncemented HA | NR | Disconnected |
| Lagerby 1998 | Cancellous screw vs. subchondral screw | NR | Disconnected |
| Sernbo 1990 | Hook pin vs. nail | NR | Subnode |
| Wihlborg 1990 | 3 pins vs. nail | NR | Subnode |
| Calder 1996 | Bipolar HA vs. unipolar HA | NR | Subnode |
| Cornell 1998 | Bipolar HA vs. unipolar HA | NR | Subnode |
| Stoffel 2013 | Bipolar HA vs. unipolar HA | NR | Subnode |
| Deangelis 2012 | Cemented HA vs. uncemented HA |  | Disconnected |
| Taylor 2012 | Cemented HA vs. uncemented HA |  | Disconnected |
| Nordkild 1985 | Nail plate vs. screw Plate |  | Disconnected |
| Figved 2009 | Cemented HA vs. uncemented HA |  | Disconnected |
| Parker 2010 | Cemented HA vs. uncemented HA |  | Disconnected |
| Parker 2010 | Long-thread screw vs. short-thread screw |  | Disconnected |
| Kim 2012 | Conventional-stem THA vs. short-stem THA |  | Disconnected |
| Eschler 2014 | Screw plate vs. screw plate |  | Disconnected |
| Keating 2005^†^ | HA vs. THA vs. osteosynthesis |  | General |
| Rogmark 2002 | Arthroplasty vs. screw or hook pin |  | General |
| Leonardsson 2010 | Arthroplasty vs. screw or pin |  | General |
| Hedbeck 2011 | Bipolar HA vs. unipolar HA |  | Subnode |
| Holmberg 1990 | Hook pin vs. nail |  | Subnode |

NR: not reported; HA: hemiarthroplasty; THA: total hip arthroplasty

^*^disconnected: we excluded trials that were not connected to the network; subnode: we excluded trials that compared interventions within the same class (eg, a trial comparing unipolar vs. bipolar HA); general: we excluded trials lacking a full description of interventions (eg, arthroplasty or osteosynthesis).

†the comparison between HA and THA in Keating 2005 was selected

References to excluded studies

1. Svenningsen, S., et al., Internal fixation of femoral neck fractures. Compression screw compared with nail plate fixation. Acta Orthop Scand, 1984. 55(4): p. 423-9.

2. Madsen, F., et al., Fixation of displaced femoral neck fractures. A comparison between sliding screw plate and four cancellous bone screws. Acta Orthop Scand, 1987. 58(3): p. 212-6.

3. Alberts, K.A., J. Jaerveus, and K. Zyto, Nail versus screw fixation of femoral neck fractures. A 2-year radiological and clinical prospective study. Ann Chir Gynaecol, 1989. 78(4): p. 298-303.

4. Skinner, P., et al., Displaced subcapital fractures of the femur: a prospective randomized comparison of internal fixation, hemiarthroplasty and total hip replacement. Injury, 1989. 20(5): p. 291-3.

5. Olerud, C., L. Rehnberg, and E. Hellquist, Internal fixation of femoral neck fractures. Two methods compared. J Bone Joint Surg Br, 1991. 73(1): p. 16-9.

6. Sorensen, J.L., J.E. Varmarken, and J. Bomler, Internal fixation of femoral neck fractures. Dynamic Hip and Gouffon screws compared in 73 patients. Acta Orthop Scand, 1992. 63(3): p. 288-92.

7. Bonke, H., Hemiarthroplasty or total hip replacement for femoral neck fractures. A preliminary report of a randomized trial. Hefte zur der Unfallchirurg, 1999. 272: p. 176-177.

8. van Dortmont, L.M., et al., Cannulated screws versus hemiarthroplasty for displaced intracapsular femoral neck fractures in demented patients. Ann Chir Gynaecol, 2000. 89(2): p. 132-7.

9. Sonne-Holm, S., S. Walter, and J.S. Jensen, Moore hemi-arthroplasty with and without bone cement in femoral neck fractures. A clinical controlled trial. Acta Orthop Scand, 1982. 53(6): p. 953-6.

10. Rehnberg, L. and C. Olerud, Fixation of femoral neck fractures. Comparison of the Uppsala and von Bahr screws. Acta Orthop Scand, 1989. 60(5): p. 579-84.

11. Emery, R.J., et al., Bipolar hemiarthroplasty for subcapital fracture of the femoral neck. A prospective randomised trial of cemented Thompson and uncemented Moore stems. J Bone Joint Surg Br, 1991. 73(2): p. 322-4.

12. Lagerby, M., S. Asplund, and I. Ringqvist, Cannulated screws for fixation of femoral neck fractures. No difference between Uppsala screws and Richards screws in a randomized prospective study of 268 cases. Acta Orthop Scand, 1998. 69(4): p. 387-91.

13. Sernbo, I., et al., Internal fixation of 410 cervical hip fractures. A randomized comparison of a single nail versus two hook-pins. Acta Orthop Scand, 1990. 61(5): p. 411-4.

14. Wihlborg, O., Fixation of femoral neck fractures. A four-flanged nail versus threaded pins in 200 cases. Acta Orthop Scand, 1990. 61(5): p. 415-8.

15. Calder, S.J., et al., Unipolar or bipolar prosthesis for displaced intracapsular hip fracture in octogenarians: a randomised prospective study. J Bone Joint Surg Br, 1996. 78(3): p. 391-4.

16. Cornell, C.N., et al., Unipolar versus bipolar hemiarthroplasty for the treatment of femoral neck fractures in the elderly. Clin Orthop Relat Res, 1998(348): p. 67-71.

17. Stoffel, K.K., et al., Does a bipolar hemiprosthesis offer advantages for elderly patients with neck of femur fracture? A clinical trial with 261 patients. ANZ J Surg, 2013. 83(4): p. 249-54.

18. Deangelis, J.P., et al., Cemented versus uncemented hemiarthroplasty for displaced femoral neck fractures: a prospective randomized trial with early follow-up. J Orthop Trauma, 2012. 26(3): p. 135-40.

19. Taylor, F., M. Wright, and M. Zhu, Hemiarthroplasty of the hip with and without cement: a randomized clinical trial. J Bone Joint Surg Am, 2012. 94(7): p. 577-83.

20. Nordkild, P., S. Sonne-Holm, and J.S. Jensen, Femoral neck fracture: sliding screw plate versus sliding nail plate--a randomized trial. Injury, 1985. 16(7): p. 449-54.

21. Figved, W., et al., Cemented versus uncemented hemiarthroplasty for displaced femoral neck fractures. Clin Orthop Relat Res, 2009. 467(9): p. 2426-35.

22. Parker, M.I., G. Pryor, and K. Gurusamy, Cemented versus uncemented hemiarthroplasty for intracapsular hip fractures: A randomised controlled trial in 400 patients. J Bone Joint Surg Br, 2010. 92(1): p. 116-22.

23. Parker, M.J. and S.M. Ali, Short versus long thread cannulated cancellous screws for intracapsular hip fractures: a randomised trial of 432 patients. Injury, 2010. 41(4): p. 382-4.

24. Kim, Y.H. and J.H. Oh, A comparison of a conventional versus a short, anatomical metaphyseal-fitting cementless femoral stem in the treatment of patients with a fracture of the femoral neck. J Bone Joint Surg Br, 2012. 94(6): p. 774-81.

25. Eschler, A., et al., Angular stable multiple screw fixation (Targon FN) versus standard SHS for the fixation of femoral neck fractures. Injury, 2014. 45 Suppl 1: p. S76-80.

26. Keating, J.F., et al., Displaced intracapsular hip fractures in fit, older people: a randomised comparison of reduction and fixation, bipolar hemiarthroplasty and total hip arthroplasty. Health Technol Assess, 2005. 9(41): p. iii-iv, ix-x, 1-65.

27. Rogmark, C., et al., A prospective randomised trial of internal fixation versus arthroplasty for displaced fractures of the neck of the femur. Functional outcome for 450 patients at two years. J Bone Joint Surg Br, 2002. 84(2): p. 183-8.

28. Leonardsson, O., et al., Long-term follow-up of replacement compared with internal fixation for displaced femoral neck fractures: results at ten years in a randomised study of 450 patients. J Bone Joint Surg Br, 2010. 92(3): p. 406-12.

29. Hedbeck, C.J., et al., Unipolar hemiarthroplasty versus bipolar hemiarthroplasty in the most elderly patients with displaced femoral neck fractures: a randomised, controlled trial. Int Orthop, 2011. 35(11): p. 1703-11.

30. Holmberg, S., et al., Fixation of 220 femoral neck fractures. A prospective comparison of the Rydell nail and the LIH hook pins. Acta Orthop Scand, 1990. 61(2): p. 154-7.

**Table C. Therapeutic classes defined by a consensus process**

| Therapeutic orientations | Therapeutic classes after concensus process | Content |
| --- | --- | --- |
| Osteosynthesis | Screw | all kinds of screws whatever the number of screws, size of screws, thread length or width |
|  | Unthreaded cervical osteosynthesis (UCO) | pin, hook pin and nail treatment, regardless of the number of implanted devices |
|  | Plate | plates and plates associated with a screw, regardless of the cervical implant |
| Arthroplasty | Hemiarthroplasty (HA) | bipolar and unipolar HA, with a cemented or an uncemented stem, regardless of the surgical approach |
|  | Total hip arthroplasty (THA) | all implant models, regardless of the size of the stem, use of cement, type of acetabular component or surgical approach |

# Figure D. Evolution of the network of the 56 trials with interventions as labeled by the original trial authors

**Table E. Summary data for each trial and for each intervention group**

|  |  | Intervention 1 | No. of events | No. of patients | Intervention 2 | No. of events | No. of patients | Intervention 3 | No. of events | No. of patients |
| --- | --- | --- | --- | --- | --- | --- | --- | --- | --- | --- |
| Sikorski | 1981 | HA | 11 | 114 | Screw | 17 | 76 |  |  |  |
| Christie | 1988 | UCO | 14 | 66 | Plate | 24 | 61 |  |  |  |
| Herngren | 1992 | UCO | 12 | 84 | Screw | 16 | 96 |  |  |  |
| Elmerson | 1995 | UCO | 43 | 122 | Plate | 40 | 100 |  |  |  |
| Jonsson | 1996 | UCO | 7 | 24 | THA | 3 | 23 |  |  |  |
| Benterud | 1997 | Plate | 42 | 108 | Screw | 34 | 117 |  |  |  |
| Johansson | 2000 | Screw | 19 | 50 | THA | 2 | 50 |  |  |  |
| Davison | 2001 | HA | 0 | 16 | Screw | 7 | 16 |  |  |  |
| Puolakka | 2001 | HA | 8 | 187 | Plate | 28 | 93 |  |  |  |
| Parker | 2002 | HA | 15 | 229 | Screw | 111 | 226 |  |  |  |
| Lykke | 2003 | Screw | 34 | 53 | HA | 7 | 47 |  |  |  |
| Roden | 2003 | Screw | 22 | 55 | THA | 1 | 55 |  |  |  |
| Tidermark | 2003 | UCO | 45 | 147 | Screw | 43 | 131 |  |  |  |
| Blomfeldt | 2005 | Screw | 10 | 30 | HA | 4 | 30 |  |  |  |
| Keating | 2005 | HA | 11 | 111 | THA | 12 | 69 |  |  |  |
| Baker | 2006 | HA | 3 | 41 | THA | 1 | 40 |  |  |  |
| Mjørud | 2006 | UCO | 23 | 98 | Screw | 27 | 101 |  |  |  |
| Frihagen | 2007 | HA | 13 | 110 | Screw | 70 | 112 |  |  |  |
| Mouzopoulos | 2007 | THA | 0 | 43 | HA | 2 | 43 | Plate | 5 | 43 |
| Macaulay | 2008 | HA | 0 | 23 | THA | 1 | 18 |  |  |  |
| Van Den Bekerom | 2010 | HA | 6 | 137 | THA | 2 | 115 |  |  |  |
| Hedbeck | 2011 | HA | 0 | 60 | THA | 3 | 60 |  |  |  |
| Watson | 2012 | Screw | 3 | 29 | Plate | 1 | 31 |  |  |  |
| Cadossi | 2013 | THA | 7 | 42 | HA | 0 | 41 |  |  |  |
| Hedbeck | 2013 | Screw | 7 | 30 | HA | 1 | 30 |  |  |  |
| Cao | 2014 | Screw | 45 | 128 | THA | 16 | 157 |  |  |  |
| Parker | 2015 | HA | 0 | 26 | Plate | 8 | 30 |  |  |  |

Events are revision surgeries; HA: hemiarthroplasty; THA: total hip arthroplasty; UCO: unthreaded cervical osteosynthesis

# Figure F. Risk of bias in 27 selected trials

**Table G. Pairwise meta-analyses for revision surgery**

| Comparison | No. of RCTs | No. of patients | OR [95%CI] | I² (%) |
| --- | --- | --- | --- | --- |
| HA vs. THA | 7 | 843 | 0.8 [0.3-2.2] | 43 |
| Screw vs. THA | 3 | 495 | 10.2 [3.1-33.1] | 57 |
| Screw vs. HA | 7 | 1119 | 7.9 [4.4-14.4] | 57 |
| Plate vs. THA | 1 | 86 | 12.4 [0.7-232.1] | NA |
| Plate vs. HA | 3 | 422 | 7.8 [3.5-17.4] | 7% |
| Plate vs. Screw | 2 | 278 | 1.0 [0.2-4.3] | 48 |
| UCO vs. THA | 1 | 47 | 2.8 [0.6-12.3] | NA |
| UCO vs. screw | 3 | 657 | 0.9 [0.6-1.2] | 0 |
| UCO vs. plate | 2 | 349 | 0.6 [0.3-1.2] | 48 |

OR, odds ratio; 95% CI, 95% confidence interval; HA: hemiarthroplasty; THA: total hip arthroplasty; UCO: unthreaded cervical osteosynthesis

# Table H. Assessment of consistency between direct and indirect evidence

| Comparison | Direct OR [95%CI] | Indirect OR [95%CI] | P value |
| --- | --- | --- | --- |
| Screw vs HA | 8.9 [4.7;17.1] | 12.4 [4.9;37.4] | 0.71 |
| Screw vs. THA | 10.1 [4.3;29.8] | 5.1 [2.1;12.6] | 0.14 |
| Plate vs screw | 1.1 [0.3;3.4] | 1.2 [0.5;3.2] | 0.45 |
| Screw vs UCO | 1.2 [0.5;2.7] | 1.7 [0.6;5.7] | 0.71 |
| Plate vs HA | 11.5 [3.9;38.5] | 11.8 [4.1;31.0] | 0.47 |
| THA vs HA | 1.6 [0.7;3.8] | 1.1 [0.4;2.8] | 0.76 |
| UCO vs plate | 0.6 [0.2;1.6] | 0.7 [0.2;2.2] | 0.43 |
| UCO vs THA | 3.1 [0.5;23.3] | 6.1 [2.5;15.6] | 0.25 |
| Plate vs. THA | 6.8 [0.8;81.8] | 9.1 [3.8;22.7] | 0.62 |

Direct and indirect estimates were derived by the node splitting method; OR, odds ratio; 95% CI, 95% confidence interval; HA: hemiarthroplasty; THA: total hip arthroplasty; UCO: unthreaded cervical osteosynthesis
